# Utilization of acid hydrolysate of recovered bacterial cell as a novel organic nitrogen source for L-tryptophan fermentation

**DOI:** 10.1080/21655979.2019.1586053

**Published:** 2019-03-18

**Authors:** Qingyang Xu, Fang Bai, Ning Chen, Gang Bai

**Affiliations:** a College of Biotechnology, Tianjin University of Science and Technology, Tianjin, China; b State Key Laboratory of Medicinal Chemical Biology and College of Pharmacy, Tianjin Key Laboratory of Molecular Drug Research, Nankai University, Tianjin, China

**Keywords:** L-tryptophan, fermentation, waste bacterial cell, acid hydrolysis, *Escherichia coli*

## Abstract

In this study, waste bacterial cell (WBC) was recovered and used as an alternative to yeast extract in L-tryptophan fermentation. The effects of sulfuric acid concentration and temperature on the hydrolysis of WBC were optimized and the amino acid content in the waste bacterial cell hydrolysate (WBCH) was increased. Plackett-Burman and Box-Behnken design analysis revealed the optimum composition of the WBCH-based fermentation medium to be 22.47 g/L WBCH, 2.26 g/L KH_2_PO_4_, and 1.25 mg/L vitamin H. L-tryptophan yield and productivity with WBCH as the nitrogen source were 52.3 g/L and 2.16 g/L/h, respectively, which were 13% and 18% higher than those obtained with the yeast extract as the nitrogen source. In addition, WBCH did not affect the growth of *Escherichia coli* during L-tryptophan fermentation. Cost accounting showed that WBCH could be used as a novel and cheap organic nitrogen source for industrial L-tryptophan production.

## Introduction

L-tryptophan, an important feed additive, plays important roles in animal nutrition balance. It promotes animal appetite, digestion, and growth, and thus is beneficial for improving animal immunity, health, and meat quality [1–3]. In addition, because L-tryptophan metabolism is closely related to many physiological functions in humans [4], researchers are increasingly exploring the use of L-tryptophan for drugs synthesis, such as antidepressants [5], alkaloids [6], and antitumor drugs through chemical or biological transformation [7].

Industrial production of L-tryptophan is currently achieved by microbial fermentation. To increase the economical efficiency of L-tryptophan production, most of the studies focused on microbial metabolic engineering and fermentation process control. During the fermentation of L-tryptophan by *Escherichia coli*, the accumulation of acetic acid caused by carbon source overflow significantly inhibits the cell growth and product synthesis [8,9]. In our previous study, we increased the production and yield of L-tryptophan by knocking-out genes encoding the key enzymes in the acetic acid synthesis pathways, including acetyltransferase (*pta*), acetate kinase (*ackA*), and pyruvate oxidase (*poxB*) [10,11]. Furthermore, we increased the specific production of L-tryptophan by optimizing the dissolved oxygen regulation strategy and employing a cell-recycle-fermentation technology [12,13].

Another factor affecting the production cost of L-tryptophan is the postprocessing of the fermentation broth (including the bacterial biomass). Currently, more than 10,000 tons of L-tryptophan is annually produced by *E. coli* in China. The production of 1 ton of L-tryptophan is accompanied by the production of 1.3–1.5 tons of waste bacterial cells (WBCs). The majority of these WBCs are directly incinerated or discharged [14], despite their known utility as animal feed or organic manure. The cell wall of the waste bacteria is mainly composed of peptidoglycan, proteins, and lipids, which can provide a variety of functional groups such as carboxyl, hydroxyl, phosphate, sulfate, and amino groups for binding of metal ions. Therefore, researchers have attempted to use the waste biomass of filamentous fungi, yeast, bacteria, or algae to remove the heavy metal ions in wastewater [15,16]. However, the use of WBC as a protein supplement or an active adsorbent is limited by its low utilization and low added value. These resources have therefore not been fully utilized.

Because there are abundant proteins, nucleotides, vitamins, and other trace elements in WBC, it can be hydrolyzed and used as an alternative nitrogen source in fermentation. Moon et al. investigated the effects of the autolysate of waste cells of *Saccharomyces cerevisiae* used for ethanol fermentation as an alternative nitrogen source in lactic acid fermentation and found that the lactic acid production, productivity, and yield increased by 17%, 41%, and 14%, respectively [17]. The endogenous autolytic enzymes in yeast cell mainly include proteases, nucleases, and glucanases; therefore, the autolysates are composed of oligosaccharides, amino acids, and nucleic acids [18,19]. In contrast, the endogenous autolytic enzymes of bacteria are predominated by muramidases, glucosaminidases, amidase and peptidase, and the autolysates are composed of oligosaccharides, N-acetylglucosamine, N-acetyl muramic acid, and oligopeptide [20,21]. Therefore, degradation of bacterial proteins into amino acids can be achieved through acid/alkaline hydrolysis or protease digestion.

In order to reuse the waste biomass and reduce the fermentation cost, in this study, we collected the WBC from an L-tryptophan fermentation broth and hydrolyzed them to obtain the waste bacterial cell hydrolysate (WBCH). The WBCH was then used as an alternative nitrogen source to yeast extract (YE) in L-tryptophan fermentation. To our knowledge, the WBCH has not been evaluated as a medium component for L-tryptophan production. We investigated the effects of different hydrolysis conditions on the total amino acid content in WBCH, and optimized an L-tryptophan fermentation medium containing the WBCH as the nitrogen source. Finally, the L-tryptophan fermentation process using WBCH as the nitrogen source was compared with that using YE as control in a 30 L fermenter.

## Materials and methods

### Microorganisms and WBCH preparation

*E.coli* TRTHBPA (*trpEDCBA*+Tet*^R^*,Δ*tna*,Δ*gltB*,Δ*pta*,Δ*ackA*) was constructed in previous study [12].

**Figure 1.**
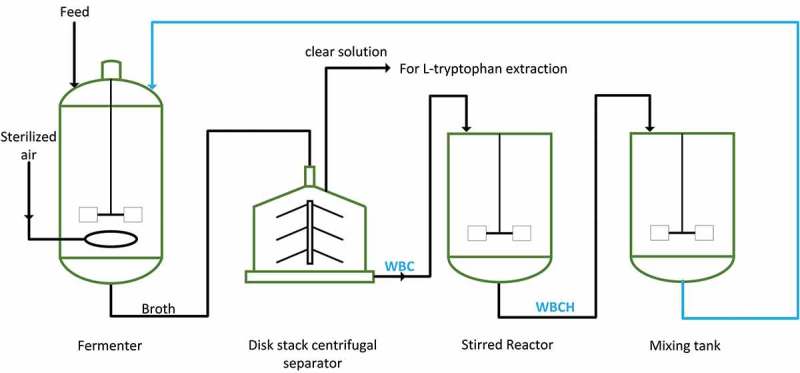
Preparation and utilization of WBCH. WBC refers to waste bacterial cell recovered by centrifugation. WBCH refers to waste bacterial hydrolysate produced by acid hydrolysis.

Preparation of the WBCH is shown in Figure 1. After L-tryptophan fermentation, the broth was continuously passed through a disk stack centrifugal separator (GEA Westfalia Separator HSD 1-06-107; Oelde, Germany). The clear solution discharged from the centrifuge was extracted to isolate L-tryptophan, and the concentrated WBC was transferred into a stirred reactor. Acid hydrolysis was conducted in the stirred reactor at a speed of 200 r/min. The mass ratio of dry WBC to sulfuric acid was 1:5. The temperature was varied from 85 to 110°C. The sulfuric acid concentration was adjusted to values ranging from 1.5 to 4.0 mol/L. WBCH was then transferred into a mixing tank and used as a nitrogen source for the next round batch fermentation.

### Medium

We used a previously reported seed medium composed of 20 g/L glucose, 15 g/L YE, 10 g/L (NH_4_)_2_SO_4_, 0.5 g/L sodium citrate, 5 g/L MgSO_4_·7H_2_O, 1.5 g/L KH_2_PO_4_, 0.015 g/L FeSO_4_·7H_2_O, and 0.1 g/L vitamin B1 [10,11]. The fermentation medium containing YE as the nitrogen source (YE medium) contained 20 g/L glucose, 1 g/L YE, 4 g/L (NH_4_)_2_SO_4_, 2 g/L sodium citrate, 5 g/L MgSO_4_·7H_2_O, 2 g/L KH_2_PO_4_, and 0.1 g/L FeSO_4_·7H_2_O.

The WBCH medium was optimized by Plackett-Burman and Box-Behnken design and analysis.

### Culture conditions

According to published procedures, seed cultures were prepared by growing cells at 36°C and 200 rpm in a 500 mL baffled flask containing 30 mL seed medium for 12 h. A 30mL inoculum of this culture was added aseptically to a 5 L seed fermenter (Biotech-2002 Bioprocess Controller, Baoxing, Shanghai, China) containing 3 L seed medium and cultivated at 36°C for 16 h [10,11]. Batch fermentations were performed in a 30 L fermenter (Biotech-2012 Bioprocess Controller, Baoxing, Shanghai, China) containing 18 L YE medium or WBCH medium. The temperature was maintained at 36°C, the pH was adjusted to 7.0 with 25% ammonium hydroxide (w/w), and the level of dissolved oxygen (DO) was controlled at 20% (0–20 h) and 30% (20–38 h). When the initial glucose was depleted, glucose solution (80% w/v) was added to the fermenter according to the DO feedback strategy [13].

### Statistical experimental design

The Plackett-Burman experimental design was used to screen the important variables of the medium that influenced L-tryptophan production by *E. coli*. [22] Based on the results of previous studies, seven variables—(NH_4_)_2_SO_4_, WBCH, sodium citrate, KH_2_PO_4_, MgSO_4_·7H_2_O, FeSO_4_·7H_2_O, and vitamin H (V_H_)—were denoted as numerical factors and investigated at two widely spaced intervals designated as −1 (low level) and +1 (high level) [10,11]. The seven variables were screened in 12 experimental runs (Table 1). The *P* value (significance level) of each concentration effect was determined by F-test. The variables with significance levels greater than 95% were considered to significantly influence L-tryptophan production.

**Table 1.** Experimental design for screening of variables using the Plackett-Burman method.

Trial	A	B	C	D	E	F	G	L-tryptophan yield (g/L)
1	1.00	1.00	1.00	−1.00	−1.00	−1.00	1.00	7.36 ± 0.06
2	−1.00	1.00	1.00	−1.00	1.00	1.00	1.00	7.95 ± 0.08
3	1.00	1.00	−1.00	−1.00	−1.00	1.00	−1.00	8.36 ± 0.06
4	−1.00	−1.00	1.00	−1.00	1.00	1.00	−1.00	9.13 ± 0.08
5	−1.00	1.00	1.00	1.00	−1.00	−1.00	−1.00	8.72 ± 0.07
6	−1.00	1.00	−1.00	1.00	1.00	−1.00	1.00	6.82 ± 0.06
7	−1.00	−1.00	−1.00	1.00	−1.00	1.00	1.00	9.16 ± 0.07
8	1.00	−1.00	−1.00	−1.00	1.00	−1.00	1.00	8.24 ± 0.05
9	1.00	−1.00	1.00	1.00	1.00	−1.00	−1.00	8.93 ± 0.06
10	1.00	−1.00	1.00	1.00	−1.00	1.00	1.00	9.25 ± 0.06
11	−1.00	−1.00	−1.00	−1.00	−1.00	−1.00	−1.00	9.01 ± 0.08
12	1.00	1.00	−1.00	1.00	1.00	1.00	−1.00	8.18 ± 0.06

± represents standard errors of the means.

The direction of steepest ascent is the direction in which a dependent variable increases more rapidly. The most efficient direction in which to move the experiment is along the line perpendicular to the contours. Typically, the line through the center of the region of interest and normal to the fitted surface is taken as the path of steepest ascent [23]. The path of steepest ascent started from the optimum point of Plackett-Burman design. To move away from Plackett-Burman design center along the path of steepest ascent, we moved 2.0, 0.2, 0.2 in the direction of WBCH, KH_2_PO_4_, and V_H_, respectively (Table 2).

**Table 2.** Experimental results of the path of steepest ascent.

Trial	WBCH (g/L)	KH_2_PO_4_ (g/L)	V_H_ (mg/L)	L-tryptophan yield (g/L)
1	20	2	1	8.85 ± 0.05
2	22	2.2	1.2	9.32 ± 0.07
3	24	2.4	1.4	9.11 ± 0.06
4	26	2.6	1.6	9.04 ± 0.06
5	28	2.8	1.8	8.76 ± 0.05

± represents standard errors of the means.

In order to maximize the response yield of L-tryptophan, the independent variables X_1_ (WBCH), X_2_ (KH_2_PO_4_), and X_3_ (V_H_) were simultaneously varied according to a Box-Behnken design (BBD) scheme. A total of 15 experiments were thus performed (Table 3). The central point of BBD was replicated thrice (experiment Nos. 4, 10, and 14). Equation (1) was calculated to estimate the effect of each independent variable on dependent variables of L-tryptophan yield. This calculation was aimed at predicting the best combination of independent variables for optimization of the reaction conditions.

(1) $$Y=b_{0}+\sum b_{i}x_{i}+\sum \sum b_{ij}x_{ij}+\sum b_{ii}x_{ii}^{2}$$

**Table 3.** Optimization of the medium components for improving the L-tryptophan yield using Box-Benhnken design.

Trial	*X*_1_ WBCH (g/L)	*X*_2_ KH_2_PO_4_ (g/L)	*X*_3_ V_H_ (mg/L)	L-tryptophan yield (g/L)
1	18	2.6	1.2	10.22 ± 0.05
2	22	2.6	1.6	11.57 ± 0.05
3	22	1.8	0.8	9.73 ± 0.05
4 (c)	22	2.2	1.2	12.50 ± 0.06
5	22	2.6	0.8	10.06 ± 0.05
6	26	1.8	1.2	10.86 ± 0.05
7	22	1.8	1.6	10.92 ± 0.05
8	18	1.8	1.2	9.18 ± 0.05
9	26	2.2	0.8	11.30 ± 0.05
10 (c)	22	2.2	1.2	12.32 ± 0.06
11	26	2.6	1.2	11.18 ± 0.05
12	18	2.2	1.6	10.80 ± 0.05
13	18	2.2	0.8	9.13 ± 0.05
14 (c)	22	2.2	1.2	12.27 ± 0.06
15	26	2.2	1.6	10.65 ± 0.05

± represents standard errors of the means.

All the statistical experiments were performed in the 500 mL baffled flask containing 30 mL reaction medium. The temperature was maintained at 36°C, and the pH was adjusted to 7.0 with 25% ammonium hydroxide (w/w) during the cultivation period.

### Analysis

Data from the statistical experiments were analyzed using Design Expert 8.0.6 software (Stat Ease Inc., Minneapolis, MN. USA). All values for L-tryptophan yield are the mean values of at least three independent fermentation procedures. L-tryptophan yield and dry cell weight (DCW) were determined as described previously [24]. Amino acid concentrations in the WBCH and YE were determined by amino acid reagent organizer (SYKAM, S-7130, Germany).

## Results and discussion

### Acid hydrolysis of WBC

YE is the most commonly used organic nitrogen in L-tryptophan fermentation by *E. coli* [10–13,24,25]. It is estimated that YE supplementation in the medium contributes about 40% of the total L-tryptophan production cost. To reduce the medium cost, ethanol fermentation waste has been served as an alternative to YE for lactic acid fermentation [17]. Vacuolar enzymes including acid phosphatase and proteinase existed in the waste biomass of *S. cerevisiae* can play an important role in the breakdown of intracellular macromolecules into nucleotides and amino acids [20]. Compared to *S. cerevisiae, E .coli* has been shown to possess enzymes that can only hydrolyze bonds in the wall peptidoglycan [21]. Therefore, the hydrolysis of intracellular protein of *E .coli* into amino acids cannot be achieved by autolysis but by acid, alkali, or proteases treatment [26].

**Figure 2.**
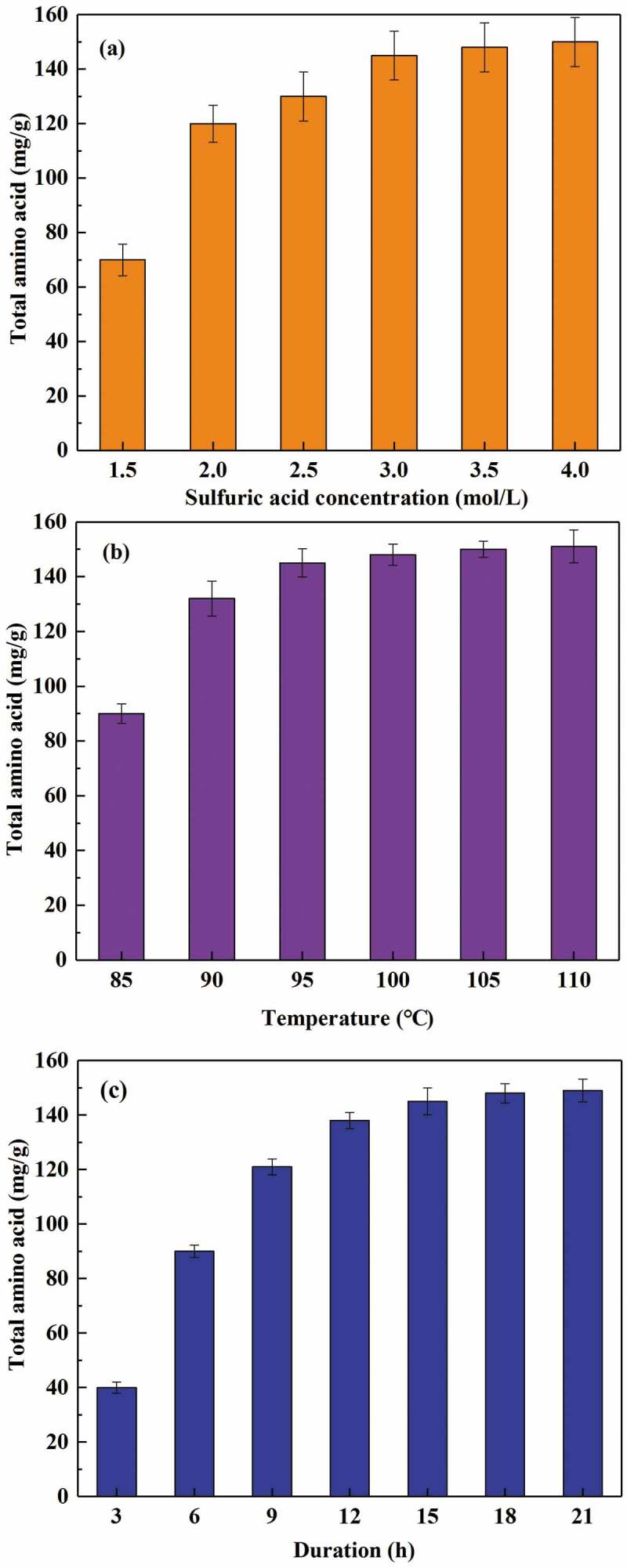
Effect of sulfuric acid concentration (a), temperature (b), and reaction duration (c) on WBC hydrolysis. The total amino acid yield is the mass ratio of extracted total amino acid to dry WBC.

We investigated the effect of sulfuric acid concentration, reaction temperature, and reaction duration on the degree of WBC hydrolysis. As shown in Figure 2, higher sulfuric acid concentration and temperature promoted protein degradation. The optimum sulfuric acid concentration and temperature for WBC hydrolysis were 3 mol/L and 95°C, which yielded 145 mg/g amino acids in the hydrolysate. Under optimal conditions, the acid hydrolysis occurred rapidly for the first 15 h, but no further hydrolysis was observed after this point. Tsugita et al. reported that peptide bonds were rapidly cleaved by a hydrochloric acid and trifluoroacetic acid mixture at a temperature of 158°C [27]. However, the utilization of volatile acids can cause pipeline corrosion, and residual trifluoroacetic acid in the hydrolysate could also affect its use as a nutrient for microbial growth. Therefore, sulfuric acid was selected as the reagent in the acid hydrolysis process, with moderate temperature and long reaction time.

### Characterization of WBCH

In order to evaluate the application of WBCH as a nutrient source in L-tryptophan fermentation, the amino acid content in the WBCH was analyzed and compared with that of commercial YE (Angel Yeast, China). As shown in Table 4, the commercial YE contained 312.69 mg/g (mass ratio of extracted total amino acids to dry yeast cell) amino acids, which was 2.15 times that in the WBC extract. The WBC extract is the water-soluble portion (mainly comprising amino acids) of the WBC obtained after acid hydrolysis, and the 14.5% recovery yield of WBC extract based on dry cell weight is in agreement with that of YE [28]. Both of YE and WBC extract contained 16 types of amino acids. Glutamate, alanine, and leucine were the main amino acids in the YE, which accounted for 18.6%, 13.1%, and 9.8% of the total amino acids, respectively. By contrast, histidine, glutamic acid, and alanine were the main amino acids in the WBC extract, accounting for 29.3%, 13.4%, and 9.7% of the total amino acids, respectively.

**Table 4.** Comparison of amino acids in YE, WBC extract, and WBCH.

Amino acid	Yeast extract/(mg/g)^a^	WBC extract/(mg/g)^b^	WBCH/(mg/g)^c^
Asparagine	16.10 ± 0.08	5.52 ± 0.08	0.86 ± 0.02
Tyrosine	14.20 ± 0.07	3.06 ± 0.07	0.48 ± 0.03
Histidine	5.50 ± 0.06	28.38 ± 0.15	4.43 ± 0.05
Proline	8.90 ± 0.05	2.22 ± 0.06	0.35 ± 0.02
Glycine	11.10 ± 0.10	11.58 ± 0.08	1.81 ± 0.02
Methionine	12.00 ± 0.09	6.54 ± 0.05	1.02 ± 0.02
Phenylalanine	12.20 ± 0.11	5.94 ± 0.06	0.93 ± 0.02
Serine	14.60 ± 0.13	2.28 ± 0.08	0.36 ± 0.03
Isoleucine	14.80 ± 0.12	3.78 ± 0.03	0.59 ± 0.01
Lysine	15.20 ± 0.11	8.94 ± 0.06	1.40 ± 0.03
Threonine	15.09 ± 0.14	10.02 ± 0.08	1.57 ± 0.02
Arginine	21.00 ± 0.16	3.18 ± 0.05	0.50 ± 0.03
Valine	22.10 ± 0.16	10.62 ± 0.09	1.66 ± 0.04
Leucine	30.60 ± 0.18	9.72 ± 0.06	1.52 ± 0.03
Alanine	41.10 ± 0.12	14.1 ± 0.06	2.20 ± 0.05
Glutamic Total	58.20 ± 0.15 312.69	19.44 ± 0.12 145.32	3.04 ± 0.06 22.72

^a^Mass ratio of extracted total amino acids to dry yeast cell. ^b^Mass ratio of extracted total amino acids to dry WBC. ^c^Mass ratio of extracted total amino acids to WBCH. ± represents standard errors of the means.

### Screening of important medium components using a Plackett-Burman design

The Plackett-Burman experiment was used to investigate the effect of different variable concentration on L-tryptophan yield. The variables were determined according to the results of previous study [10,11]. Biotin (V_H_) was selected as a variable because the biotin in the WBC was damaged during acid hydrolysis. When the sign of the concentration effect of the tested variable is positive, the influence of the variable upon L-tryptophan yield is greater at a higher concentration, and if it is negative, the influence is greater at a lower concentration. The significance level of the tested variable was determined by the F-test (Table 5). Three variables B (WBCH), D (KH_2_PO_4_), and G (V_H_) had confidence levels above 95% (Pr > F value less than 0.05) and were considered to significantly influence L-tryptophan production by *E. coli*.

**Table 5.** F-test and significance levels calculated from Plackett-Burman experiments.

Trial	Factors (g/L)	Level		*F* value	Pr > F	Significance
		Low (−1) High (+1)				
A	(NH_4_)_2_SO_4_	4	5	0.17	0.7052	NS
B	WBCH	20	25	29.98	0.0054	*
C	Citric acid	2	2.5	1.84	0.2460	NS
D	KH_2_PO_4_	2	2.5	6.51	0.0432	*
E	MgSO_4_·7H_2_O	2	2.5	5.10	0.0869	S
F	FeSO_4_·7H_2_O	0.15	0.188	0.76	0.4316	NS
G	V_H_ (mg/L)	1	1.25	9.43	0.0373	*

### Path of steepest ascent

The path of steepest ascent was used to determine the direction of concentration changes of the significant variables (WBCH, KH_2_PO_4_, and V_H_) to improve L-tryptophan production [29]. As shown in Table 2, L-tryptophan yield was the highest when the reaction medium contained 22 g/L WBCH, 2.2 g/L KH_2_PO_4_, and 1.2 mg/L V_H_. The result suggests that the selected variable concentration was near the region of maximum yield response.

### BBD and response surface analysis

In order to determine the best medium composition for maximum L-tryptophan yield, we used BBD to investigate the effect of significant variables (WBCH, KH_2_PO_4_, and V_H_) based on the results of Plackett-Burman experiment (Table 3). The central point of BBD (experiment Nos. 4, 10, and 14) was designed based on the results of path of steepest ascent. The second-order polynomial equation for L-tryptophan yield in terms of coded factors was derived as follows:

(2) $$Y=12.46+0.58X_{1}+0.29X_{2}+0.46X_{3}-0.18X_{1}X_{2}-0.58X_{1}X_{3}+0.08X_{2}X_{3}-1.10X_{1}^{2}-1.00X_{2}^{2}-0.89X_{3}^{2}$$

Where Y is the predicted L-tryptophan yield, *X*_1_ is WBCH concentration, *X*_2_ is KH_2_PO_4_ concentration, and *X*_3_ is V_H_ concentration. The significance of Equation (2) was checked by F-test, and the analysis of variance for response surface quadratic model is summarized in Table 6. The model *P* value of 0.0024 indicates the model was significant. The lack of fit *P* value of 0.1902 indicates that ‘Lack of Fit’ was not significant relative to the pure error. The goodness of the model was checked by the determination coefficient of the regression equation. The adjusted *R*^2^ of Equation (2) was 0.9595, indicating that only 4% of the total variation could not be explained by the model.

**Table 6.** Variance analysis of regression equation of Box-Benhnken design.

Factor	Sum of squares	Degree of freedom	Mean square	F value	Pr > F
Model	16.29	9	1.81	18.76	0.0024*
X_1_-WBCH	2.71	1	2.71	28.15	0.0032*
X_2_-KH_2_PO_4_	0.68	1	0.68	7.10	0.0447*
X_3_-V_H_	1.73	1	1.73	17.94	0.0082*
X_1_X_2_	0.13	1	0.13	1.34	0.2987
X_1_X_3_	1.35	1	1.35	13.95	0.0135*
X_2_X_3_	0.026	1	0.026	0.27	0.6284
X_1_^2^	4.48	1	4.48	46.47	0.0010*
X_2_^2^	3.70	1	3.70	38.42	0.0016*
X_3_^2^	2.94	1	2.94	30.44	0.0027*
Residual	0.48	5	0.096		
Lack of fit	0.42	3	0.14	4.41	0.1902 (NS)
Pure error	0.063	2	0.032		
Total variation	16.77	14			
*R*^2^ = 0.9712			Adjustment *R*^2^ = 0.9595		

**Figure 3.**
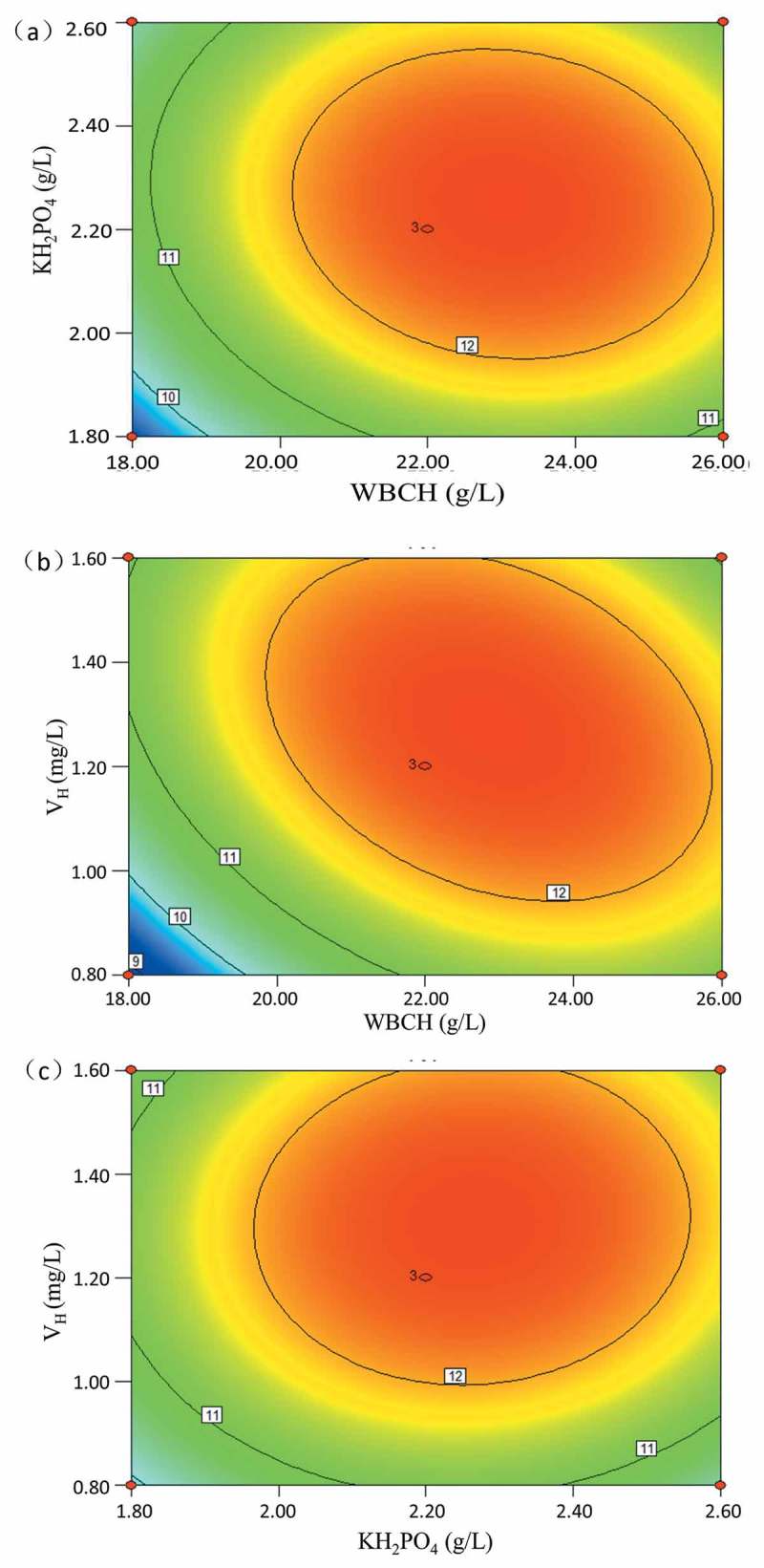
Combined effect of (a) WBCH and KH_2_PO_4_ concentration; (b) WBCH and V_H_ concentration; (c) KH_2_PO_4_ and V_H_ concentration on L-tryptophan production.

The significance of each coefficient of Equation (2) was also determined by the F-test. As shown in Table 6, the coefficients *X*_1_, *X*_2_, *X*_3_, *X*_1_^2^, *X*_2_^2^, *X*_3_^2^, and *X*_1_*X*_3_ were significant model terms because their Pr > F values were less than 0.05. These results suggest that the selected variables and some of their interactions had significant influence on L-tryptophan production. The contour plots of the combined effects of each variable are shown in Figure 3. It was found that L-tryptophan yield was the most sensitive to the combined effects of WBCH and V_H_. As biotin is an important coenzyme for microbial growth, it has been used widely for induction of amino acid production [30]. Therefore, the WBCH medium needs biotin supplementation because the WBCH lacks biotin. At constant KH_2_PO_4_ concentration, simultaneous increase in the concentration of WBCH and V_H_ (experiment Nos. 1 and 2), increased the L-tryptophan yield from 10.22 to 11.57 g/L.

The predicted optimal medium composition for L-tryptophan production was 22.47 g/L WBCH, 2.26 g/L KH_2_PO_4_, and 1.25 mg/L V_H_. In order to confirm the optimization results, L-tryptophan fermentation was performed using the predicated medium composition. The average result from three replicates (12.61 g/L) was consistent with the predicted value (12.59 g/L), and the model was proven to be adequate.

### Performance of the WBCH medium and YE medium in the 30 L fermentation

**Figure 4.**
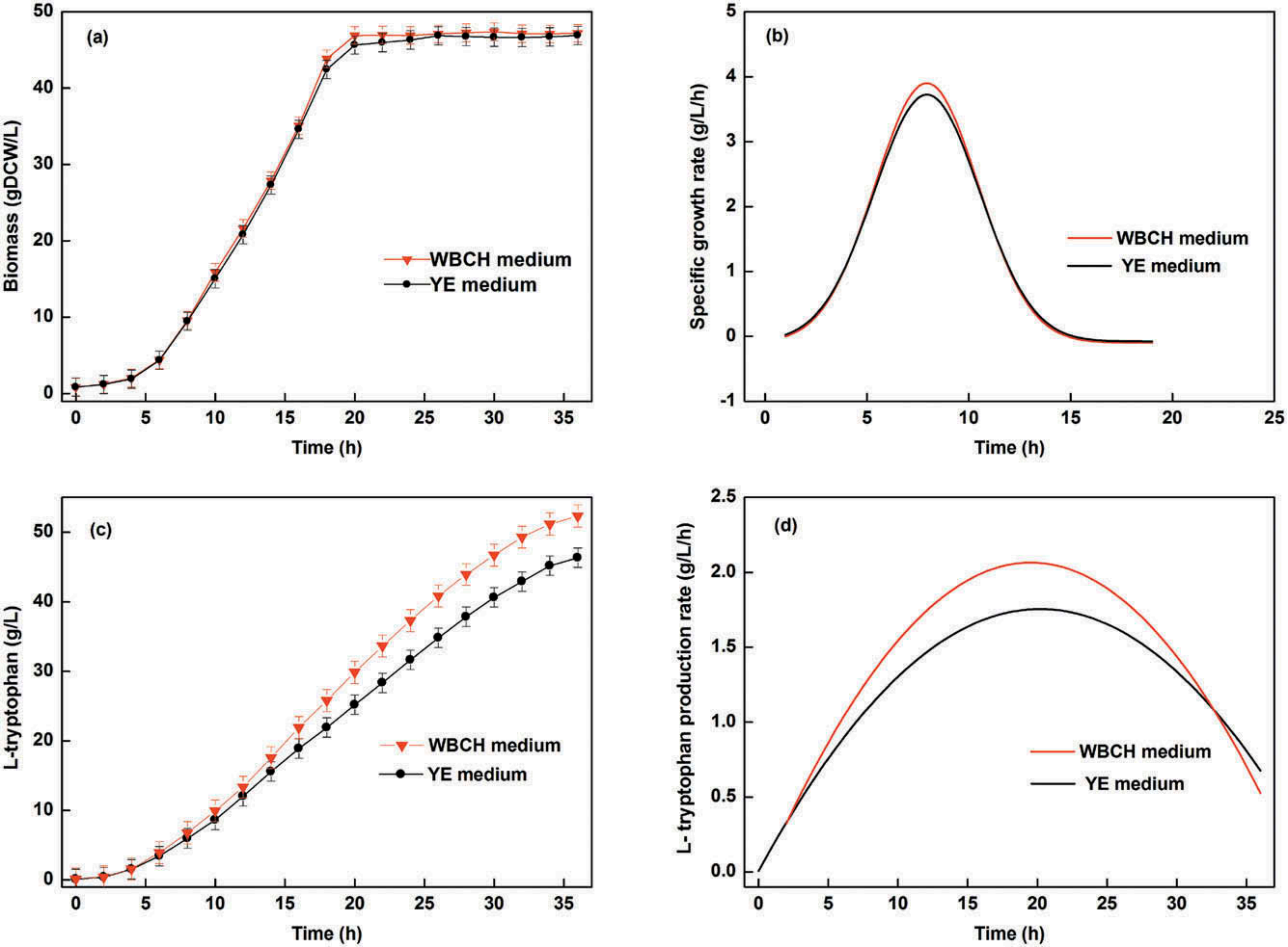
Time courses of (a) biomass formation; (b) specific growth rate; (c) L-tryptophan titer; and (d) L-tryptophan production rate by *E. coli* using WBCH medium and YE medium.

The fermentation yield using the optimized WBCH medium and a YE medium was compared in a 30 L fermentation. As shown in Figure 4, the final L-tryptophan titer and productivity obtained using the WBCH medium were 52.3 g/L and 2.16 g/L/h, which were 13% and 18% higher than those obtained using the YE medium, respectively. Furthermore, the biomass concentration of *E. coli* during L-tryptophan fermentation using the WBCH medium and the YE medium was almost the same. Because WBCH was obtained by acid hydrolysis, the residual sulfuric acid was neutralized by ammonium hydroxide during fermentation. The resulting (NH_4_)_2_SO_4_ can be assimilated by *E. coli* and used as a quick-release nitrogen source. Therefore, higher maximum specific growth rate of *E. coli* was found in the WBCH medium. In addition, differences in the composition of the WBCH and YE medium may affect the microbial activity and metabolic state, which could also explain the improvement in the specific growth rate and L-tryptophan production rate in the WBCH medium.

### Comparison of the fermentation costs using the WBCH medium and the YE medium

To evaluate the feasibility of using the WBCH as an alternative nitrogen source for L-tryptophan production, the cost of WBCH was calculated based on the production method (Table 7). To produce 1 kg WBCH, 0.16 kg WBC, 0.23 kg sulfuric acid, and 0.06 kg ammonium hydroxide were needed. In addition, the consumption of steam and electricity for WBCH production was 0.07 and 0.04 kg, respectively. According to the market price in China, the total production cost of 1 kg WBCH was￥0.67 RMB. Thus, the total cost of the organic nitrogen source in the WBCH medium was￥15.1 RMB/kL, which 40% of that in the YE medium (￥38.0 RMB/kL, the YE price was offered by Angel company). Moreover, after acid hydrolysis, the WBCH can be added directly to the fermentation medium without sterilization. Based on these findings, use of the WBCH as the organic nitrogen source can dramatically decrease the nutrient and sterilization costs of L-tryptophan fermentation. Moreover, the wastewater and waste biomass treatment costs can also be reduced by using this efficient cell recycling technology.

**Table 7.** Calculation of production cost of WBCH.

Materials	Consumption	Unit price (RMB)	Cost(RMB/kg)
WBC	0.16 (kg)	2.0	0.320
Sulfuric acid	0.23 (kg)	0.6	0.138
Ammonium hydroxide	0.06 (kg)	2.0	0.12
Steam (0.8 MPa)	0.07 (kg)	0.08	0.056
Electricity	0.04 (KW·h)	0.8	0.032
Total			0.666

## Conclusion

In the present study, WBC produced in L-tryptophan fermentation was recovered, hydrolyzed, and reused as a novel organic nitrogen source for the next round of L-tryptophan fermentation. Our results showed that increasing the sulfuric acid concentration and temperature during hydrolysis can increase the amino acid content of the WBCH. By statistical analysis and optimization, the best composition of the WBCH-based L-tryptophan fermentation medium was found to be 22.47 g/L WBCH, 2.26 g/L KH_2_PO_4_, and 1.25 mg/L V_H_. The final L-tryptophan titer and productivity in a 30 L fermentation using the WBCH medium were 13% and 18%, respectively, higher than those obtained using the YE medium. Reuse of WBC can effectively reduce the cost and waste of L-tryptophan production.
